# Conversations upon the Physical and Mental Hygiene of Girlhood

**Published:** 1881-09-20

**Authors:** T. S. Powell

**Affiliations:** Atlanta; Professor of Obstetrics and Diseases of Women, and Lecturer on Medical Ethics in the Southern Medical College


### Southern Medical Record:

EDITORS:

T. S. POWELL, M.D. W. T. GOLDSMITH, M.D. R. C. WORD, M.D.

*R. C. WORD, M.D., Managing Editor.*

☞All Communications and Letters on Business connected with the RECORD must be addressed to the Managing Editor.

VOL. XI. ATLANTA, GA., SEPTEMBER 20, 1881. NO. 9.

### ORIGINAL AND SELECTED ARTICLES.

### CONVERSATIONS UPON THE PHYSICAL AND MENTAL HYGIENE OF GIRLHOOD.

BY T. S. POWELL, M. D.,

Professor of Obstetrics and Diseases of Women, and Lecturer on Medical Ethics in the Southern Medical College.

#### CONVERSATION IX.

Mother—"Good morning, Doctor, I hope you are in a mood to be catechized to-day. I have been so anxious to resume that last subject: I had just asked you an important question."

Doctor—"What was that, madam?"

Mother—"How we could get the full confidence ot our children so they would have no secrets from their parents."

Doctor—"My dear madam, by precept, example and *close companionship* with your children. I think that fathers and mothers should be *associates* of their children as long as both live. To fill this place towards their sons and daughters, parents must enter into all their feelings, sympathize with them in their joys and sorrows, listen kindly and affectionately to their every grief and perplexity, however small it is, and encourage them in every way to talk freely to their parents about everything in which they are interested."

Mother—"Yes, I see, Doctor, that is an excellent idea. By making ourselves companions for our children, we would get nearer to them than in any other way."

Doctor—"Yes, madam; and if all parents and children were thus associated you have no idea how much moral, mental and physical danger would be averted in the lives of girls and boys, and how much usefulness and happiness might be insured for both children and parents."

Mother—"Doctor, I think we mothers ought to talk to our young daughters about our own early lives, tell them our love scrapes, episodes, etc., and let them see and feel that they are not different from their mothers."

Doctor—"You are correct in that, madam; and when it is done in the proper way, and connected with appropriate counsel and advice, will doubtless be of much advantage to young girls; it will assist them in guiding their judgment in such affairs when they are called to act a part in them. And by the way, I do not think a child's will in anything should be crushed or 'conquered,' as is usually said, but, instead, the will should continually be *guided* into the right direction. This should be done with kindness yet firmness, by proper information, and by your own example. A child, as soon as it is old enough to understand, should be told the reason for things, why this is best and that is not, and what will be the result in both cases, and show it how to make the test and the application."

Mother—"Yes, Doctor, and I am sure this would be much better than to cruelly punish a child until its will is broken, its spirits crushed and cowered, and its whole nervous system almost thrown into convulsions, and——"

Doctor—"Its health damaged by this intense nervous excitement. But when the other plan is pursued, this physical injury will be avoided, and I do not think that either girls or boys will then have secrets from their mother, and she will be saved many heartaches in regard to their health and morals; for I cannot conceive of a woman being such a monstrosity of moral nature as to ever give her child bad advice upon any subject."

Mother—"Doctor, do you think we can control our children in regard to the books and literature they read?"

Doctor—"You certainly can, madam, if you have your children's entire confidence. I do not think they would then even look into a book or newspaper without first bringing it to you to know if it was suitable for them to read. And, if a mother is what she ought to be, and can be, the girls or boys in such cases, will cheerfully abide by her decision, and will not try to read the book in secret, because they have been trained to feel that their mother is truly their friend—that she knows what is best for them; and would not deny them any pleasure that would do them no harm."

Mother—"And does not this objectionable literature have a bad effect upon the health of girls? Of course, I know it does upon their morals."

Doctor—"Yes, madam, and this is also an important consideration involved in the health of young girls. If it is necessary for the physical health of children and young people that they should have pure, nutrititious and unstimulating food, that for the mind should be of the same. Sensational novels highly seasoned, and impossible love stories, tales of vice, murder and other crimes, all have a deleterious and often fatal effect upon the health of young girls. They excite to a feverish extent, the passions and emotions, create fears, horrors and depression from forebodings of evil in the mind, all of which affect the physical condition."

Mother—"And yet the majority of our young people read scarcely any other kind of books and newspapers."

Doctor—"Very true, madam, but as a professional man it is my opinion that no such literature should be read by girls or boys until they are eighteen years old, if it is ever read at all. I have often heard of insanity in young people, caused by habitual reading of such books and newspapers. One medical author goes so far as to say that this literature, and also balls and theatres, should be forbidden fruit to every young person and prohibited as positively as strychnine or arsenic, not even allowed as subjects of discussion or argument."

Mother—"I presume he thinks that introducing girls and boys to the customs and amusements of society gives a premature excitement to the emotional nature, which is injurious to health."

Doctor—"Yes, madam, and he is right to this extent, such indulgence tends greatly to force a premature womanhood, and at a time when with her 'exquisitely frail and delicate organization, all made of nerves and sensibility, the young girl is the most impressionable of human beings.' The demands of these customs and amusements must necessarily greatly tax her physical powers, besides destroying that maidenly freshness and innocence which is the chief charm of young womanhood, and as the same medical author asserts, that at the pace we are going in society, this charm will soon have no existence, except in the dreamy visions of poets and romancers."

Mother—"Yes, I think it is indeed a girl's greatest attraction, is better than mere physical beauty and all the artificial graces of society."

Doctor—"The proper control and cultivation of the temper, passions, and emotions are inseparably connected with the perfection of physical education, and all are continually subject to unhealthy excitement and wrong directions in what is called fashionable society, especially when the young girl is introduced to it at her most susceptible unguarded age. If after a few years she escapes with a moderate degree of health, a moral defloration has taken place, that leaves an impress upon her tastes, inclination and principles, and which will perhaps remain through life."

Mother—"Doctor, do you think our boarding schools for g rls, and also for boys, are what they ought to be?"

Doctor—"Well, madam, that question is rather broad in its extent, but if I were to give you a condensed answer in the words of a noted professional man who has had much personal observation of the subject, I would tell you that he solemnly declares he would sooner have his boys live in a small-pox or plague hospital than in any one of the boarding schools he attended himself when a boy, and such as one to which he sent his own little son for a few months."

Mother—"Is it possible, Doctor? Why, it is horrible! And did not this physician enquire about the character of the school before he sent his little boy there?"

Doctor—"He certainly did, and as the institution was of a professedly religious and family character, presided over by a clergyman, and received pupils of only first class families, the father thought it a model school. But during his little boy's first vacation at home, he made revelations in regard to the vice and immorality, taught and practiced by the pupils, but, of course unknown to the teachers, which, made the father shudder to hear. If he had not very properly trained his boy to have no secrets from his parents, he would have remained at the school, and no doubt, been ruined in body and soul."

Mother—"Oh! Doctor, this is distressing. It is dreadful to hear, but surely there is no danger in our female boarding schools?"

Doctor—"I cannot say there is no danger at any of them. You know that if there is only one bad girl in a school, she can either corrupt or give some taint of her wickedness to all with whom she associates. Here is where teachers should be of a character and temperament to win the confidence of their pupils so that they will not be too timid to make known to their instructors any secret violation of morality or rules of the school."

Mother—"Yes, I am sure that would soon lessen the evil, and finally obliterate it, if the teachers acted properly upon the information."

Doctor—"The mere existence of a boarding school is not responsible for its errors and vices, it is neglect in conducting it properly and with a thorough and ceaseless vigilance of every pupil, accompanied by a kind, impartial but rigid authority."

Mother—"Yes, I think that word impartial is very well put in, for I have no doubt pupils are often taken into boarding schools, and kept there when they are known to be thoroughly bad, because the teachers do not wish to lose the wealthy patronage of their parents."

Doctor—"No doubt that is sometimes the case. The hygienic features also as regards plenty of fresh air, proper warmth, wholesome food and exercise, superintendence of dress, etc., are very deficient in the great majority of these schools."

Mother—"A lady friend told me not long since that her health was so injured at a female college, her parents were compelled to take her home after her first term of tuition."

Doctor—"How was that, madam?"

Mother—"She said the winter was bitter cold, and she and her three room-mates had to study two hours every evening until bed time, but they never had a good fire in their room the whole winter. The servant would put green, hard wood in the grate, without sufficient kindling, and the fire would go out in a few moments; so the girls had to set there at study, shivering with cold, and then go to bed in that condition. The lady said one turn of this green wood would stay in the fireplace a whole week before it burned out, although kindling was put under it every night and morning."

Doctor—"I am not surprised that the girls' health was injured, but I hope and believe that these evils will soon be done away with in all our schools of the best reputation. If boys and girls cannot be educated at a good home, a good school is the next best place, and when it is necessary to send them from home, much of the danger to their health and morals could be avoided if parents would previously teach their children properly about all this neglect, imprudence and immorality that may be found at the school, and warn them of the consequences, and charge it upon them that if any of this is practised or permitted in the school, to inform their parents of it without delay."

Mother—"And then they would be called tale-bearers, and perhaps be ill-treated for 'telling tales out of school'."

Doctor—"Then, madam, the children should also inform their parents of this, and the latter should at once remove them from the school. That old saying "you must not tell tales out of school" will not do for this progressive age, and it must have been invented and enforced by teachers and pupils whose conduct would not bear close investigation at all times."

Mother—"Yes, I think so. But, Doctor, so few parents are educated so they can really know when their children are doing well at school, either mentally, morally or physically, and many of them do not even think of it."

Doctor—" Tben, my dear madam, before they attempt to superintend the proper education of their children they should go to school themselves, or gain the necessary information at home, by reading the books that treat of these subjects in a plain, practical manner."

Mother—" If all children could have the influence of a good home, and be educated there, it would be better."

Doctor—" Yes, madam, undoubtedly, but all children cannot have these advantages since many of them are obliged to be sent from home to be educated. If all parents and instructors would teach their children the plain moral maxim that " even an evil thought, when encouraged, is a sin committed; that if the evil thoughts come they must not be welcomed and entertained," it would be a shield and defense for boys and girls against the approaches of the many evils incident to modern education."

Mother—" I heard a lady friend who is a hopeless invalid say, not long ago, that if her mother had told her all these things, and especially guarded her against reading sensational novels and plays, which she did read before she was scarcely in her teens, and that if she had known how to take care of her health in every respect before it was too late, she would have given all the wealth of the world for this knowledge."

Doctor—" I have no doubt she would, and have been glad to make the exchange, if she could have seen the results as she now sees them."

Mother—" As she is, this lady is a most admirable woman in every respect, and if she had had this knowledge in time, I believe she would have been nearly perfect in body and mind, and would have lived a long life in perfect health, and the highest state of earthly happiness."

Doctor—" There are many such sad cases, my dear madam, and yet mothers are too timid, or have too much false delicacy, to give their daughters these instructions upon which so much depends, but they " put into the hands of these daughters the journals of the day, though they are teeming with advertisements and news items of the most revolting and indecorous character." I long for the time to come when our mothers and daughters will intelligently discriminate between real delicacy of feeling and principles and the sham article so much used."

Mother—"It does seem difficult, Doctor, for us to do that."

Doctor—"But it can be done very easily, my dear madam, if mothers will properly educate themselves and their daughters, and try to cultivate the pure and womanly heart which is always quick to discern between the real good and the real evil."

Mother—"And will follow the one and shun the other."

Doctor—"Certainly, I think, madam, that not only women should know how to take care of their own health and happiness, and look after that of their husband and children, but young men should be sufficiently informed to watch over the health of the woman he intends to marry, so far as the proper decorum and delicacy will permit."

Mother—"I am glad you have mentioned that, Doctor, for I have had a similar opinion, but did not know if I was right "

Doctor—"But you were right, madam, for when the young girl becomes the wife of the young man, he will then know how to assist her in preserving her health and vigor, which is of so much importance to the health and happiness of both. His sympathy will be ever ready for her sufferings, and her headache, and her hysterical burst of tears will not drive him from his home, feeling that his wife is cross and unreasonable, but he will know she is really suffering from her nerves being all out of tune, and he will also know the best course to pursue on such occasions, instead of becoming cross and irritable himself, which will only increase his wife's nervous disorder and perhaps bring on an attack of illness."

Mother—"I wish, Doctor, all husbands could hear you say that, for while many of them wish to be kind, and are really well-meaning, they do not know how to manage these disorders to which nearly all women are subject, and which appear to be little things, but are really painful and of much importance."

Doctor—"You are right, madam, and we now see that while a mother should make it a daily practice to remark the countenance and manner of her children, to ascertain if they are feeling quite well and comfortable, the husband should also observe that of his wife, and satisfy himself that her movements and general appearance indicate health, strength and vivacity. Many wives and daughters are overworked, and become diseased on account of it, while the husband and father does not seem to be conscious of their condition, and makes no effort to relieve it when it is in his power to do so."

Mother—"Yes, I know an excess of exercise is hurtful as well as too little."

Doctor—"In my professional visits to some families, I often see the strength of young girls exhausted by doing too much house-work, at one time—'cleaning up,' as they call it. When they are through with it they drop into a seat, pale, exhausted, and with all the brightness and buoyancy of youth taken out of them by overtaxed physical strength. The mothers also, have an habitual weary look as if continually under a heavy burden, and it is in just such constant conditions of the system in mothers and daughters that disease frequently sows its seeds of invalidism or death. When exercise is taken just sufficiently to be healthful, it gives color instead of paleness, and strength instead of exhaustion."

Mother—"Doctor, how many hours sleep out of twenty-four do you think children of different ages should have?"

Doctor—"Well, madam, if all adults should have seven or eight hours sleep to preserve vigor of body and mind, and elasticity of spirits, of course children should have more. If infants sleep two-thirds of the time, it is so much the better; from two to six years children should sleep at least twelve hours out of twenty-four; from six to fourteen, not less than ten hours, and then on to eighteen both boys and girls should have at least nine hours sound sleep."

Mother—"But to get the early morning air, in fine weather, after this amount of rest, the children would have to retire very early."

Doctor—"Yes, madam, and that is what they should do. But in our large towns and cities, children, young girls and boys as well as adults, are up entirely too late at night. This dissipation among the wealthy classes, overwork among the lower, and among farmers wives, added to unwholesome food, and miserably prepared at that, and entire ignorance of all laws of health, will destroy the vigor of any woman, and make her look old and jaded at an age when she ought to be fresh, blooming, and retaining all her youthful vivacity."

Mother—"It seems cruel to me to even awake children and invalids or sick persons from a sound, refreshing sleep."

Doctor—"And it never should be done, unless absolutely necessary. As I have just said, if children and all persons retire to rest at the proper time, if well, nature will awake them when they have had sufficient sleep, provided they are not disturbed by outside causes."

Mother—"Do you not think, Doctor, that it injures the health of children and women to sleep in a room or any place, where they suffer from fear, whether there is really any cause to be afraid or not?"

Doctor—"It certainly does, madam. It is apt to bring on painful, nervous disorders in women, and I have known girls and boys made subject to convulsions because they were compelled to sleep in places where they felt excessive fear every night."

Mother—"I am now quite satisfied, Doctor, that before parents can inaugurate a system for rearing perfectly healthy and happy girls and boys, we must first train ourselves in the proper school of knowledge. I think you intimated that no girl or young man should be considered fit to enter the marriage state until he or she is thoroughly acquainted with the laws of life and health, so they will be capable of preserving their own health and moral tone, and their children's, and thus be able to promote the comfort and happiness of their families."

Doctor—"Yes, madam, that is the idea, and we cannot fail to see the importance and necessity for this advanced step in civilization when we look at the great number of unhappy, ailing children, and the actual slaughter of these innocents as the annual death rate in our cities and other communities give a startling evidence."

Mother—"Yes, that is as sad as it is true. If all mothers knew how to save their babes and little children from the dreaded approach of disease and death, how many desolate homes would now be full of joy."

Doctor—"And this knowledge is not so difficult of attainment, my dear madam, as the uninformed might suppose. These laws, though of exquisite complication are so perfectly arranged and adapted to each other they are easily comprehended when correctly taught, and their harmony of adaptation is so apparent and beneficent, we find that nature's mandates, as is said of wisdom in the Scriptures, are ways of pleasantness and peace."

Mother—"Yes, Doctor, they are indeed, when we allow nature to execute her laws, and in her own perfect manner."

Doctor—"Yes, and while learning and obeying these laws, every woman who does so, is unconsciously becoming that most beneficent benefactor of the human race—a sanitary reformer. I have here an extract from a lecture by Dr. Richardson, before the English Sanitary Congress, and you will see what a high estimate he puts upon these possible attainments of women."

Mother—"Read it, Doctor, if you please. I am sure it will give me some good, perhaps new ideas."

Doctor—"The Doctor observed that long before sanitation was heard of every good, cleanly housewife was a practical sanitary reformer. The office of the prevention of disease was especially fitted for women. The training required was simple, and every woman willing to go through it might become by it, mistress of the destinies of the world. She should master physiology so as to understand the general construction of the human body, and know the great systems of the body—the digestive, the ciculatory, the respiratory, the nervous, the sensory, the absorbant and glandular, the muscular, the oseous or bony and the membraneous. If she would act on this knowledge, there would hardly be one deformed child left in the land in one or two generations."

Mother—"Such a result as that alone, Doctor, would be well worth the time and care in acquiring the knowledge."

Doctor—"It would indeed, madam. But the doctor further says, that one effort on the part of woman, as a sanitarian, would call forth all her powers. She would stand to resist with her full persuasive might that process which I have elsewhere described as the intermarriage of disease. She will tell her sisters what that process means, that diseased heredity united in marriage means the continuance of the heredity almost as certainly as that two and two make four; that insanity, consumption, cancer, scrofula, yes, and certain of the contageous diseases also, may be perpetuated from the altar; and that the first responsibilities of parents toward their offspring ought to be, not how to provide wealth and position, over which they have no control, but that preliminary, healthy parentage, which is the foundation of health, and without which position and wealth are shadowy legacies indeed. The doctor here says that he may be answered that this is delicate ground upon which to tread."

Mother—"But while that is true, Doctor, when the subject is so very important, it cannot be wrong to go over the ground with proper reverence."

Doctor—"No, madam, surely not, and as Dr. R. continues, when we are living in a world where those who study the living and dead most carefully, very seldom see a man or woman hereditarily free from disease, even this ground must be entered upon by the enlightened scholar. "I touch upon it here," he says, "for the best of all reasons, that the subject it includes, effecting deeply the human heart in its sympathies and affections, is one upon which the influence of woman, the arbitress of the natures that are to be, is all-potent for good or for evil. To know the first principles of animal physics and life; to learn the simpler problems relating to the fatal diseases, to ordain the training of the young, and to grasp the elements of the three psycho-physical problems—the human temperaments, the moral contageons with their prevention, and the heredities of disease with their prevention, are earnestly and respectfully proposed as the heads of the educational programme for our modern woman in her sphere of life and duty."

Mother—" And do you not think, Doctor, that a woman can have what is necessary of this important information and yet retain all her purity and delicacy of feeling, and womanly qualities just the same. ?"

Doctor—"Certainly she can, my dear madam. To be instructed in the elements of this knowledge I think will really add to the purity and reverence of a woman's nature. The study of any of God's works cannot promote impurity in the heart of his creatures, if that study is pursued as it always should be, in a reverent spirit; and woman, even more than man, is impressed by the goodness, the purity and wisdom of the Creator, reflected in all his works."

Mother—" Yes, I think she is, and she receives the teachings of nature more readily than man accepts them, without question, soon as she sees how strikingly nature's laws are illustrated in herself, and in her children. I have gained a good deal of valuable information in these talks, Doctor, of which you have been so kind as to give me the benefit, and I am sorry they are about to come to a close. But from what I have learned, I know I will be better fitted to rear my children than I ever have been before, and I hope to be benefitted so much by your instructions and what I yet expect to gain from all available sources, I will become before it is too late, not a doctor, no indeed, but a wife and mother in the truest and fullest sense of the word."

Doctor—"And I believe you will, my dear madam. You have a right conception of your duty and your opportunities, and I believe you will attain what you so earnestly desire."

Mother—"Thank you, Doctor, I hope I will. If, by the help of God I can rear my sons and daughters after the truest models of moral and physical perfection, I know I will have an abundant reward in their love and gratitude, and also in that of my husband, for preserving his children in health, beauty and happiness. Then they will be a great joy and blessing to us when old age comes on, if it pleases God to spare our lives till then."

Doctor—"And, my dear madam, you will also have the gratitude of every intelligent, truly philanthropic man and woman, as every mother does have, who thus rear her children, for they prove not only a great blessing to their parents, but to the whole community in which they live, and to the world at large."

Mother—"Yes, that is very true. I do not know how to thank you, Doctor, for what I have learned from your teachings as well as for the restoration of my daughter's health."

Doctor—"Then do not try, my dear madam. Our profession should always be an office for doing good; it is the dearest ambition of the true physician, and to know that he has the heartfelt gratitude and just appreciation of those he tries to benefit gives him a happy compensation, and makes green spots of refreshment along his laborious way. Miss Mary, I am glad you have come in time to say good-bye, and for me to remind you of our bargain. You are to be obedient to your mother's counsel and mine, and keep those red cheeks and that bright complexion. As you will soon be ready for the social circle and at the proper time enter upon the responsibilities of womanhood, I will call and have a talk with your mother and yourself on what constitutes the true woman."

Mother—"You are so kind. I would like so much to have your views on that subject. Don't forget to come, Doctor."

Doctor—"No, madam, I will not forget. Good morning; good bye, Miss Mary, "remember" is the watch word."

